# Sport federation investment in health promotion: program implementation and viability: Aurélie Van Hoye

**DOI:** 10.1093/eurpub/ckac131.341

**Published:** 2022-10-25

**Authors:** A Van Hoye, C Regan, A Lane, B Cullen, A Vuillemin, C Woods

**Affiliations:** 1 Physical Education and Sport Sciences, University of Limerick, Limerick, Ireland; 2 Community and Health Department, Gaelic Athletic Association, Dublin, Ireland; 3 SHE Research Group, Technological University of the Shannon, Athlone, Ireland; 4 Innovation and Research, Sport Ireland, Dublin, Ireland; 5 LAHMESS, Université Côte d'Azur, France, France

## Abstract

**Background:**

Researchers have called for a better recognition of the potential of sports clubs for health promotion (HP), but less is known on the support provided by sports federation. The present study analyses the the implementation of the Gaelic Athletics Association (GAA) Healthy Club Project, to investigate its organization’s viability.

**Methods:**

A single case study was realized, based on observation, document analysis and interviews, to document the viable system model.

**Results:**

Results have identified a three-level structure, where 6 employees at national level support the work of 28 volunteer’s county health and well-being officers and 439 clubs implicated. Strengths of the organization are the identification of a single national referent for clubs or county, the learning process and openness to novelty, as well as the enhanced workforce through county implication as role model. Challenges are the financial and human resources provision, the ability to implicate county and club board and the training in HP of volunteers. Interlevel relationship are supported by the creation of a community of practice and the centralization of the project at national level, but hindered by a clear definition of county tasks. The strengths of the controlling system include a steering committee implicating partners completing each other and the proper use of evaluation to build evidence on the project, where challenges remains in wider collaboration within the GAA.

**Conclusions:**

The present work has underlined key scaling up factors of the HCP implementation to support its viability, which could be learnt from other sports federation implementing HP interventions.

**Key messages:**

• Sports federation have a key role to play to support sports clubs to promote health, by developing specific programs.

• Health Promotion development in sports clubs has similar scaling up implementation factor than other public health interventions.

